# Immunological Monitoring to Rationally Guide AAV Gene Therapy

**DOI:** 10.3389/fimmu.2013.00273

**Published:** 2013-09-12

**Authors:** Cedrik Michael Britten, Steffen Walter, Sylvia Janetzki

**Affiliations:** ^1^Translational Oncology, University Medical Center, Johannes Gutenberg-University Mainz (TRON gGmbH), Mainz, Germany; ^2^Association for Cancer Immunotherapy (CIMT), Mainz, Germany; ^3^Immatics Biotechnologies GmbH, Tübingen, Germany; ^4^ZellNet Inc., Fort Lee, NJ, USA

**Keywords:** immunological monitoring, assay harmonization, gene therapy, adeno-associated virus, biomarkers

## Abstract

Recent successes with adeno-associated virus (AAV)-based gene therapies fuel the hope for new treatments for hereditary diseases. Pre-existing as well as therapy-induced immune responses against both AAV and the encoded transgenes have been described and may impact on safety and efficacy of gene therapy approaches. Consequently, monitoring of vector- and transgene-specific immunity is mandated and may rationally guide clinical development. Next to the humoral immune response, the cellular response is central in our understanding of the host reaction in gene therapy. But in contrast to the monitoring of antibodies, which has matured over many decades, sensitive and robust monitoring of T cells is a relatively new development. To make cellular immune assessments fit for purpose, investigators need to know, control and report the critical assay variables that influence the results. In addition, the quality of immune assays needs to be continuously adjusted to allow for exploratory hypothesis generation in early stages and confirmatory hypothesis validation in later stages of clinical development. The concept of immune assay harmonization which includes use of field-wide benchmarks, harmonization guidelines, and external quality control can support the context-specific evolution of immune assays. Multi-center studies pose particular challenges to sample logistics and quality control of sample specimens. Cooperative groups need to define if immune assessments should be performed in one central facility, in peripheral labs or including a combination of both. Finally, engineered reference samples that contain a defined number of antigen-specific T cells may become broadly applicable tools to control assay performance over time or across institutions.

## Introduction

Inherited diseases account for a substantial number of hospital admissions of children but also affect adults. Despite progress in science, efficient treatments that go beyond supportive care are not available for most of these diseases. Gene replacement therapies bear a high potential to address the medical need in affected patients and have been introduced to clinical testing about 20 years ago (1). After many draw-backs in the past an increasing number of reports of successful gene therapies in patients with hemophilia (2), Leber’s congential amaurosis (LCA) (3), or X-linked adrenoleukodystrophy (4) were recently published. In November 2012, Glybera^®^ (alipogene tiparvovec) was the first human gene therapy to receive a market approval from the European Medicines Agency (EMA) suggesting that the field has reached a turning point which may give raise to additional approved gene therapies in the future (5).

Most strategies applied for gene replacement focus on two types of vectors to reach stable transfer of functional gene products into selected target tissues, namely lentiviral vectors for *ex vivo* gene transfer into hematopoietic cells or other stem cells, or adeno-associated virus (AAV) for *in vivo* gene transfer to various target tissues such as muscle, the liver, the retina, lung, or the brain. Despite successes reported for AAV gene therapy approaches it has become clear that humoral and cellular immune responses against both the vector as well as the transgene may negatively impact on safety and efficacy of gene therapy approaches (6). Humoral responses against AAV frequently occur in the population, increase by age and are efficiently induced following a single administration of AAV-based therapies. Neutralizing antibodies against AAV have been shown to negatively influence transduction rates and may counter-act therapy approaches with systemic delivery of AAV in particular. More recently, pre-existing antigen-specific T cells recognizing AAV capsid proteins have been suggested to be an independent factor leading to reduced transduction rates on one hand and immune-mediated clearance of transduced cells expressing capsid proteins on the other hand, which led to immune-related adverse events in patients (7, 8). The first results showing that human CD8^+^ T cells to AAV capsid could limit the efficacy of the gene transfer originated form an initial trial of AAV gene transfer to the liver for treatment of hemophilia (7). Here it could be shown that expression of the initially expressed factor IX (F.IX) disappeared after several weeks, accompanied by a transient elevation of transaminases of the liver that were shown to be mediated by antigen-specific T cells directed against AAV but not the therapeutic F.IX that eliminated vector-transduced hepatocytes. In addition, extensive T cell monitoring from multiple studies performed in gene transfer to the muscle indicate that there is a dose dependent increase in treated patients showing T cell reactivity against the AVV capsid. Interestingly, currently available data suggest that gene transfer to immune-privileged body compartments such as the eye and central nervous system so far did not lead to detectable immune responses to the capsid or the transgene. In summary, the immunogenicity data found in the clinical studies conducted with AAV-based vectors in human and available in literature, confirm that immune responses against AAV capsid proteins can vary widely and amongst others are influenced by the target organ, route of delivery, and dosing schedule (9). Also antigen-specific T cells against the encoded therapeutic gene have been described and may reduce efficacy of gene therapy (10). All these findings explain why approaches to better understand or block immune responses following gene transfer with viral vectors have recently come into focus in the field (11).

Adeno-associated virus-specific T cells have been shown to be directed against AAV capsid proteins inducing transient hepatitis following i.v administration of AAVs targeting the liver (7). Although transient toxicity may be acceptable for regenerating tissues such as the liver in regard of safety, immune-mediated removal of AAV-transduced cells may become a major obstacle for gene transfer into toxicity relevant organs such as the brain or the heart. AAV-specific immunity may also lead to safety concerns as well as efficacy loss in replacement of the retinal pigment epithelium-specific 65-kDa protein gene in the retina in patients with LCA as re-therapy in the other eye may be mandated (12).

The currently available data on AAV-therapy induced immunity supports the notion that detailed knowledge of the presence of antigen-specific T cells prior to gene therapy as well as the occurrence of vector- and transgene-specific immunity following therapy may guide clinical decision making in the future (6, 9). Examples for proactive use of immunological data for the benefit of patients could be: (i) the selection of patients that have a high likelihood to exert the wanted effects, (ii) identification of patients that may need an adaption of the therapy (e.g., lower doses, improved vector), or (iii) receive concomitant immune-suppression until immunogenic capsid proteins are cleared. It may also be used to (iv) identify patients at risk for adverse immune reactions following second administration of the vector, or (v) be early indicators of loss of function of the encoded gene product.

**Figure 1:**
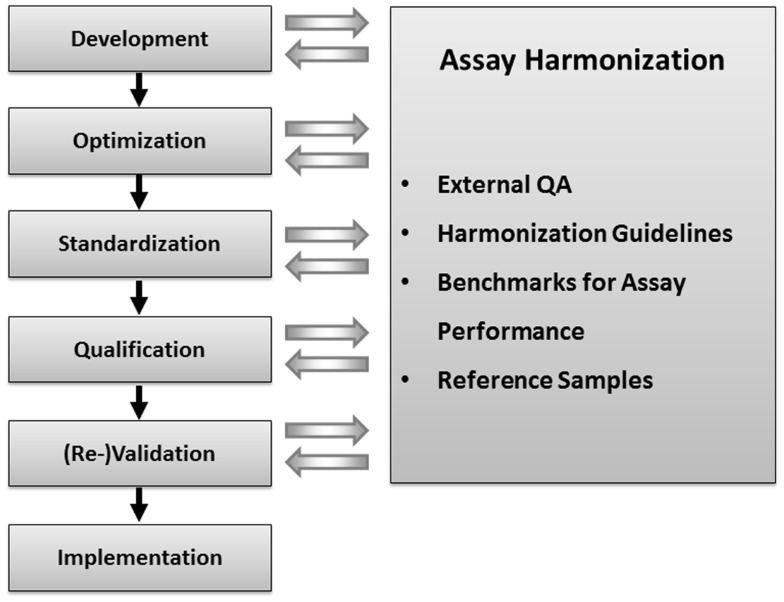
**Concept of immune assay harmonization: external quality assurance, harmonization guidelines, benchmarks for assay performance, and reference samples with defined numbers of functional antigen-specific T cells can be used to control the performance of immune assay in one lab**. Immune assay harmonization can support quality of immune assays at any step of assay development and use, starting from the initial assay development until assay validation.

In order to confirm the hypothesis that data generated by immunological monitoring can indeed impact on clinical decision making to enhance AAV-mediated gene therapy, more systematic analyses of AAV-specific T cell immunity are mandated and have even been recommend by NIH Recombinant DNA Advisory Committee (13). The fact that T cell assays may have a higher complexity and variability as compared to assays to quantify soluble analytes explains why all investigators that perform correlative biomarker studies should know, control and report on the variables of assay conduct that are known to critically affecting immune assay performance. The critical variables were identified by large scale proficiency programs conducted by the Cancer Research Institute’s Cancer Immunotherapy consortium (CRI-CIC), the Cancer Immunotherapy Immunoguiding Program (CIMT-CIP) (14), and the Human Immunophenotyping Consortium (15). In addition analytical labs are advised to optimize and standardize sample logistics and assay conduct prior to testing specimens from clinical trials even at the earliest stage of clinical development in which hypothesis generation represents the primary aim of immune assay use. Assay qualification and validation become mandatory requirements when a hypothesis generated in exploratory research from early clinical development has to be confirmed in advanced clinical development (16). This gradual evolution of immune assays from first exploratory use toward validated assays performed under Good Clinical Laboratory Practice (GCLP) standards may be supported by assay harmonization at any stage (Figure 1).

## Immune Assay Harmonization and Variables that Impact on Assay Performance

A plethora of assays exists to evaluate specific T cell responses, wanted or unwanted. These assays differ in their sensitivity to detect low frequency T cells, quantity of information obtainable, ability to determine structural and/or functional features of T cells, and their complexity in qualification and validation demands (17). For years the field of T cell monitoring has been plagued by a seemingly inherent variability in assay results, even between assays evaluating the same sample in the same laboratories or by different laboratories. A divergence of Standard Operating Procedures (SOPs), the introduction of new reagents and tools by manufacturers, preferences by the assay performing scientist, varying availability of equipment or the qualification, and training status of personnel, has further complicated the issue. Due to the lack of stringent QC procedures as required for clinical diagnostic tests, many assay “cooks” brewed their own results “soups” following their recipe without knowledge of how good or how bad that soup “tasted.” An additionally complicating fact is the limited availability of test samples, and the intrinsic biological variability in performance of such samples, a factor well known today that has caused a shift in considerations concerning sample handling for T cell monitoring (18–20).

This heterogeneous landscape of assays and protocols exists in cancer immunology, infectious diseases, transplantation immunology, and gene therapy. As outlined earlier a multitude of gene therapy approaches exist that (i) address several unrelated diseases, (ii) apply various viral vectors and serotypes, (iii) target different tissues (e.g., liver, muscle, retina, central nervous system), (iv) are applied using different administration routes and (v) deliver various therapeutic gene products. The fact the gene therapy comes in different flavors mandates a product specific adaption of the immunological monitoring that acknowledges therapy-related peculiarities and integrates available knowledge on immunogenicity and epitope hierarchy of both the applied vectors and therapeutic transgenes. The fact that there is not one single assay format that is applicable for all gene therapy trials poses a challenge on the gene therapy community to agree on some standards that may be used across a variety of clinical products and studies to generate results that may be comparable across institutions.

**Figure 2:**
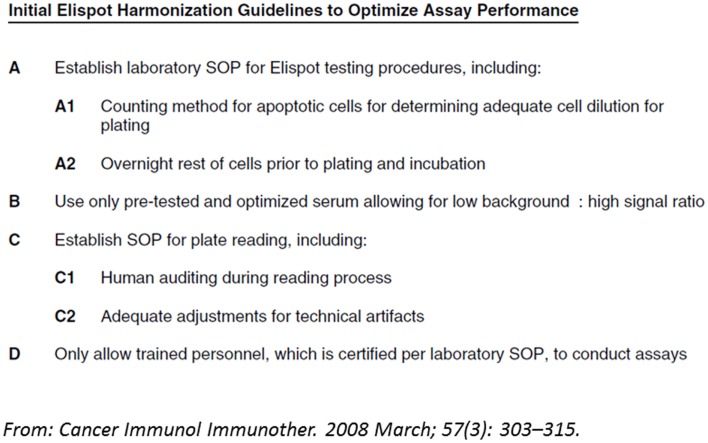
**Example of a harmonization guideline: initial guidelines for harmonization of the ELISPOT assay to optimize assay performance and reproducibility derived from two international proficiency panels, based on their findings and trends observed**.

First activities examining the differences in T cell assay performance between laboratories evolved more than 10 years ago from cancer immunologists and researchers in the HIV field that initiated proficiency panel projects for ELISPOT (21, 22). This concept was soon adopted by two non-profit organizations from the cancer research field, the CIMT-CIP and CRI-CIC, and elevated to highly efficient programs that address proficiency testing of assay performance of individual labs and the identification of variables influencing the assay outcome at large (14). By sending out pretested samples in a blinded fashion to a large number of laboratories which had to test those sample with a given T cell assay following their own protocol, and report the results back to a central site including details about how they performed the assay, CRI-CIC and CIMT-CIP were able to not only give feedback on the performance of each individual laboratory in comparison to the other participating laboratories, but also identified critical protocol variables that influence the results reported back. These studies indicated that independent of SOPs applied, the results of a significant proportion of labs accumulated around an overall median value in results, while others were outliers. With the help of protocol information provided, initial recommendations for SOP adaptations were deduced and introduced to the field for the next testing round, where it could be confirmed whether these recommendation can indeed improve performance. This iterative testing process allowed the identification of protocol steps that, independent of the SOP applied, could elevate the assay performance of labs (23–26). These findings are summarized in harmonization guidelines which are now increasingly used by laboratories (Figure 2). The introduction of such harmonization guidelines has led to significant improvement in response detection across laboratories (27) and decreased the overall variability in results between laboratories. Further investigations of specific recommendations have led to even more detailed guidance in the harmonization process, for example by investigating the use of serum-free freezing and assay media in ELISPOT (28, 29), the implementation of a dump channel and viability marker in MHC-peptide multimer staining (26) and obtaining gating recommendations for flow-based experiments (30, 31). Such assay harmonization activities, consisting of: (i) a mechanism for regular external proficiency testing with fast feed-back loops to participants, (ii) dynamic harmonization guidelines and (iii) benchmarks for assay performance, can support investigators to improve and maintain quality of their assays at any stage of development (Figure 1).

While this assay harmonization process is ongoing and an increasing number of labs apply its results, there exists a discrepancy between what is being done in laboratories, and what is reported when publishing results from T cell assays. To address the lack of transparency often observed in publications that hinders the accurate interpretation and replication of results, as well as the comparison of results from different laboratories, the Minimal Information about T cell assays (MIATA) project was introduced (32). A 3-year extensive public consensus process involving more than 100 scientists from the different fields of translational immunology and regulatory agencies as well as editors from scientific journals resulted in a final reporting framework for results of T cell assays (33) that summarizes the minimal information to report about assay conduct. Easy implementation tools are available online (miataproject.org), and MIATA-compliant papers are listed with a link to the original publication.

These activities, together with available harmonization guidelines, if followed, elevate the T cell immune monitoring field to a level that allows the creation of high quality data that support immunotherapeutic developments and advances, and hence deserve the embracement by the translational science community at large.

## Immune Assays in Multi-Center Clinical Trials

A further complicating factor for performing T cell immunomonitoring is the complexity and fragility of the analyte. In most cases, analysis is focused on T cells obtained from peripheral blood; less often (as more invasive sampling is required) T cells infiltrating other tissues such as bone marrow or skin are analyzed.

Importantly, functional T cells assays are sensitive to the time passed since the sample has been taken on a typical scale of hours (18, 19, 34). In terms of assay validation language, the sample stability is low. This can be both influenced by the inherent instability of the T cells and by the instability of the sample matrix such as granulocytes that become activated over time (35). For some assay parameters, this time window may be prolonged by adding stabilizers or by isolating the T cells without cryopreservation prior to shipping the sample (36). More generally, there are two known solutions to this logistical challenge: (i) all measurements are being performed within short time using fresh samples, or (ii) all T cells are isolated from the biological samples and cryopreserved before the assay.

The first solution may be especially attractive in the case of monocentric studies. A major drawback of such a scenario is that no retrospective analyses will be possible, longitudinal samples of a patient cannot be analyzed within the same assay, and in the case of multi-centric clinical trials this requires all immune assay parameters to be fully standardized at each site where patients are recruited. Therefore, this solution that has been termed as “peripheral analysis” (37, 38) poses high demands on assay standardization, which should be completed before a trial is initiated. Nevertheless, using fresh samples does not control inter-assay variability, and hence can introduce variability in measurements of samples obtained from different time points from a patient, which can confound the response determination between time points. Determining the precision between measurements of samples obtained from different time points is logistically challenging, hence the introduced degree of variability by testing fresh samples cannot be easily defined.

The second solution requires isolation and cryopreservation of the T cells shortly after the sample has been taken. Once the cells are frozen and kept at cryogenic temperatures, they are stable for very long time periods and can be shipped over great distances to enable batch-wise analysis within one central lab, thereby reducing assay variation. This approach does control variability by allowing the simultaneous testing of samples obtained from different time points in one assay. However, isolating and cryopreservation of T cells from biological samples is a relatively laborious process and is not performed as part of the tests for clinical routine. Therefore, if it has to be implemented within a multi-centric clinical trial, all aspects of blood collection, transport to the laboratory isolating T cells, conduct of the isolation and cryopreservation process at the lab and shipping of the frozen sample should be standardized between labs using fully comparable processes and materials (39). This solution has been termed as “central analysis” (37, 38) and poses high demands on standardized sample collection, while allowing more flexibility on the assay that are later being performed with the collected samples. Recent harmonization efforts have addressed cryopreservation challenges, and provide some guidance (28), while others are still ongoing. The performance of cell isolation and cryopreservation may be monitored by counting viable cell numbers at the time of isolation and after thawing the samples and in the case of blood samples the yield can be additionally monitored by comparing the numbers of isolated cells to the numbers of lymphocytes and monocytes from a routine hematology assessment.

To demonstrate the reproducible performance of immunoassays over time in one lab during the conduct of a clinical trial, and between labs as in the case of peripheral analysis, a stable source of reference samples that can be repeatedly analyzed is of central importance. For T cell assays, the generation of a stable reference sample is a challenging task compared to chemical assays, for example, where a small molecule can be spiked into a sample matrix at pre-defined concentrations. In the past, labs have often prepared local reference samples by aliquoting and cryopreserving cells from one large blood draw that contained T cells of a known reactivity. Such reference samples are limited in size, not comparable between different batches, and do not contain pre-defined “known” numbers of T cells. Recently, a first generation of reference samples that contain a defined number of functional antigen-specific T cells have been developed by CIP (40). The technology is based on transfer of antigen-specific T cell receptors (TCRs) into primary lymphocytes using viral gene transfer. Second generations of reference samples that are based on the use of *in vitro* translated RNA for TCR gene transfer are under development and has been shown to be simple to manufacture, robust, and sensitive, stable of at least 1 year and shown to be suitable for ELISPOT assays, HLA-peptide multimer staining as well as cytokine flow cytometry applications (unpublished data). A completed proficiency panel with 12 labs in Europe and the USA showed that the technology is easily transferable to other labs and across protocols. Given the currently available data on T cell reactivity against the AAV capsid, a set of reference samples engineered with capsid-specific TCRs for the most prevalent HLA-restrictions may become a useful tool for the gene therapy community and help to control assay performance and increase comparability across different studies using AAV gene transfer. Thus RNA-based TCR-engineered reference samples may become a standard tool to control immune assay performance over time and help destigmatizing T cell assay use in clinical trials an become yet another component of immune assay harmonization.

## Conclusion/Summary

Systematic immune assessments in gene therapy trials using quality controlled immune assays will help to understand the immunological interactions between AAV-based therapies and the adaptive immune system. Associating immunological biomarker data with data on clinical safety and efficacy will enable the field to use immunomonitoring data for clinical decision making to improve gene therapy approaches. Prior to use of immune assays investigators need to know and control those assay variables that determine the quality of sample specimens and assay results and should provide structured reporting on the assay setup in publications and reports. By providing a fast feedback of assay performance over time or across institutions, assay harmonization can support immune assay development and use and thus may be considered as a tool to enhance biomarker research and development of new immune- and gene-therapies.

## Conflict of Interest Statement

The authors declare that the research was conducted in the absence of any commercial or financial relationships that could be construed as a potential conflict of interest.
